# A case of advanced gastric cancer achieved a pathological complete response by chemotherapy

**DOI:** 10.1186/s40792-017-0344-9

**Published:** 2017-05-12

**Authors:** Kazuhiro Tada, Tsuyoshi Etoh, Yuki Shitomi, Yoshitake Ueda, Manabu Tojigamori, Hidefumi Shiroshita, Norio Shiraishi, Masafumi Inomata

**Affiliations:** 1 Department of Gastroenterological and Pediatric Surgery, Oita University Faculty of Medicine, Hasama-machi, Idaigaoka 1-1, Oita, 879-5593 Japan; 2 Center for Community Medicine, Oita University Faculty of Medicine, Oita, Japan

**Keywords:** Pathological complete response, Long-term survival, Advanced gastric cancer

## Abstract

**Background:**

Although chemotherapy is the first recommended treatment of unresectable gastric cancer, a pathological complete response is a rare event.

**Case presentation:**

A 58-year-old male was diagnosed as gastric cancer with a bulky tumor, lymphadenopathy, and suspicious peritoneal dissemination. The patient underwent chemotherapy with S-1 and cisplatin. After three courses of chemotherapy, a computed tomography showed dramatic improvements in gastric wall thickening, shrinkage of lymphadenopathy, and disappearance of disseminated peritoneal lesion. The patient underwent potentially curative resection by total gastrectomy with D2 lymph node dissection. Histological examination revealed the absence of malignant cells not only in the resected specimen but also in the harvested lymph nodes. At present, more than 7 years after the initial surgery, the patient is still alive without any recurrence.

**Conclusions:**

We obtained a pathological complete response by chemotherapy with S-1 and cisplatin for advanced gastric cancer. Although a pathological complete response is a rare event, it would be associated with the long-term survival of patients with advanced gastric cancer.

## Background

Gastric cancer is the second leading cause of cancer-related deaths worldwide. The prognosis of gastric cancer with distant metastasis is extremely unfavorable because of only 15% chance of survival for the next 5 years [1]. According to the guidelines, chemotherapy is the first recommended treatment of unresectable gastric cancer based on clinical trials [2, 3]. The clinical response rate reported for chemotherapy for unresectable advanced or recurrent gastric cancer is 28–54% [4–9]. Oral 5-FU was considered gold standard until recently S-1 + cisplatin (SP) replaced it [2, 4]. Several novel combined chemotherapy regimens occasionally have good response for unresectable gastric cancer and subsequently allow potentially curative gastrectomy [10–12]. However, a pathological complete response (pCR) is a rare event even if these powerful regimens are used.

Herein, we demonstrate a case achieved a pCR by chemotherapy with S-1 in combination with cisplatin regimen followed by surgery for advanced gastric cancer.

## Case presentation

**Fig. 1:**
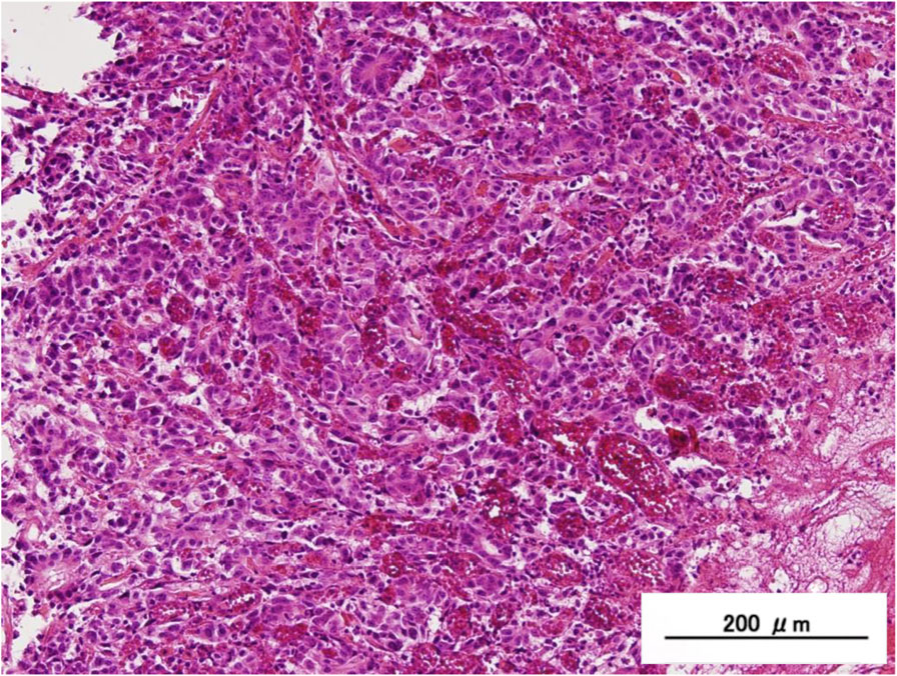
Histological findings by hematoxylin and eosin staining. Pathological examination of biopsy specimen revealed moderately differentiated tubular adenocarcinoma

**Fig. 2:**
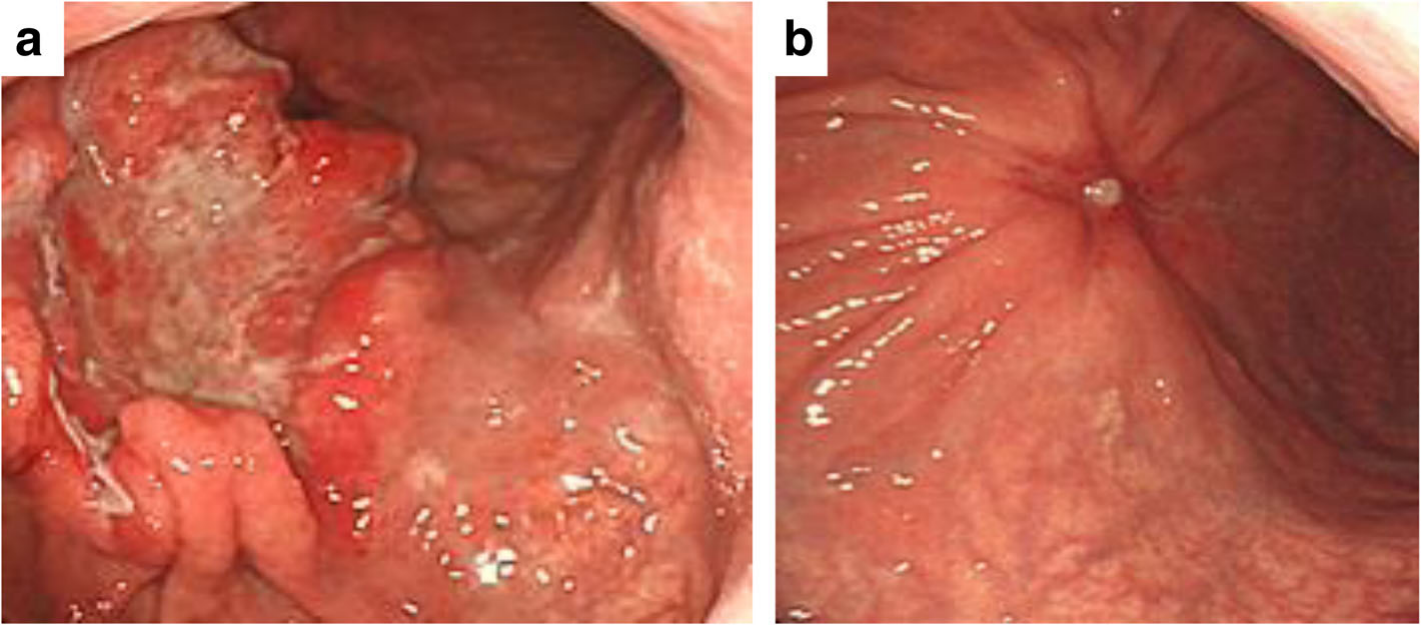
Macroscopic findings by upper gastrointestinal endoscopy. **a** A type 3 tumor measuring 95 × 75 mm in diameter was identified in the anterior wall of the gastric corpus. **b** After three courses of chemotherapy, remarkable shrinkage in tumor size was observed

**Fig. 3:**
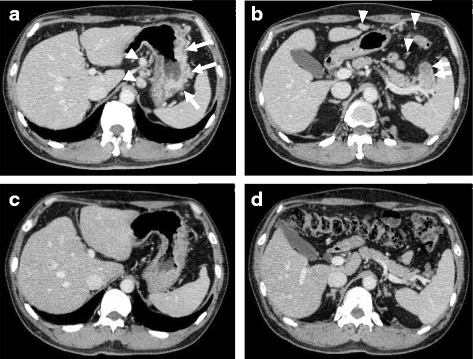
Computed tomography findings. **a** Thickening of the gastric wall, high density of fat around the gastric wall (*arrow*), and perigastric lymphadenopathy (*arrowhead*). **b** Perigastric lymphadenopathy (*arrow*) and multiple disseminated lesions (*arrowhead*). **c** Improvement of gastric wall thickening after chemotherapy. **d** Shrinkage of lymphadenopathy and disappearance of disseminated lesions after chemotherapy

**Fig. 4:**
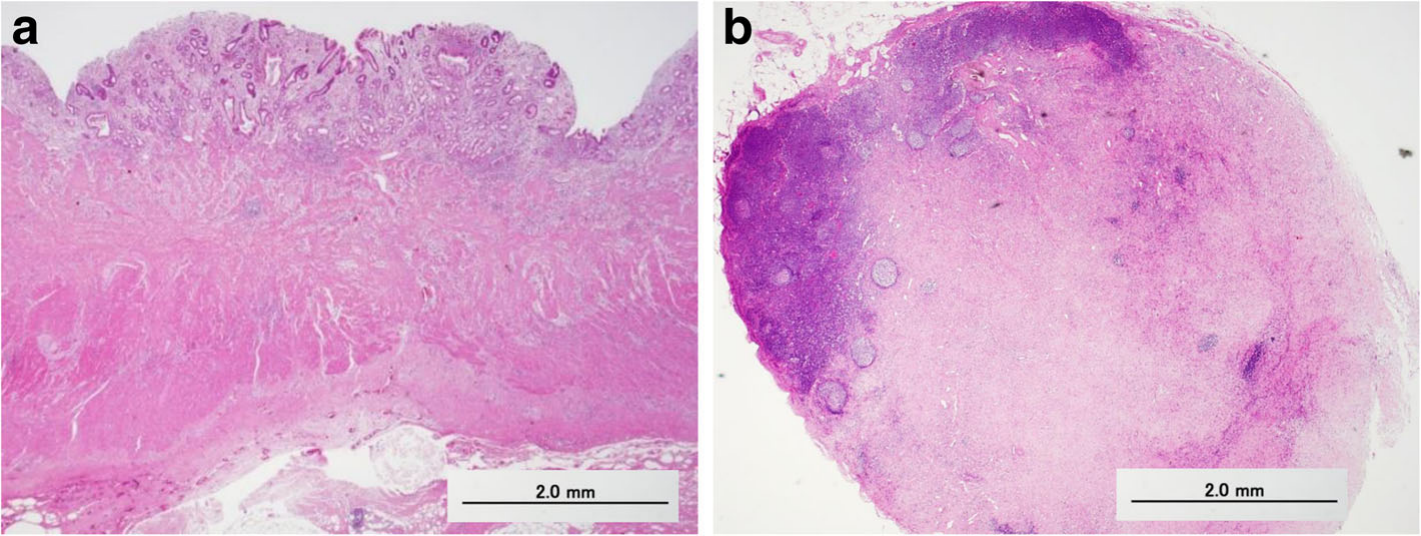
Histological findings by hematoxylin and eosin staining. **a** Malignant cells had disappeared in the resected stomach. **b** Fibrotic tissue was found to have replaced the cancer tissue in the dissected lymph node

A 58-year-old male suffering from epigastralgia was referred to our hospital. Upper gastrointestinal endoscopy revealed a type 3 tumor, measuring 95 × 75 mm in diameter in the anterior wall of the gastric corpus. Pathological examination of biopsy specimen revealed moderately differentiated tubular adenocarcinoma (Fig. 1). Computed tomography (CT) showed gastric wall thickening, and a high density of fat around the gastric wall, suggesting tumor infiltration into the gastric serosa. In addition, perigastric lymphadenopathy and multiple nodules in the upper peritoneal cavity were observed. These findings confirmed that the diagnosis of this patient as advanced gastric cancer with suspicious peritoneal dissemination. We diagnosed this patient as T4aN3M1(P). The tumor markers were within normal levels. Then, the patient underwent chemotherapy with the following treatment regimens, considered as one course: S-1 (120 mg/day, days 1–21) and cisplatin (90 mg/body, day 8). After three courses of chemotherapy, upper gastrointestinal endoscopy revealed remarkable tumor shrinkage (Fig. 2a, b). In addition, CT revealed a notable improvement in gastric wall thickening, shrinkage of lymphadenopathy, and disappearance of disseminated lesions in the peritoneal cavity (Fig. 3a–d). Because of the partial response according to the Response Evaluation Criteria in Solid Tumors (RECIST ver 1.1) [13], we aimed to perform potentially curative resection after obtaining informed consent from the patient. The diagnosis at the time was T3N1M0. No disseminated tumors and only scar were observed intraoperatively, and the patient successfully underwent potentially curative resection by spleen-preserving total gastrectomy with lymph node dissection of the splenic hilum (No.10). Histological examination of the resected specimen and the harvested lymph nodes revealed that a pCR was achieved because no malignant cells were found (Fig. 4a, b). Furthermore, cytological examination provided no evidence of cancer cells. The postoperative course of the patient was uneventful. The patient received S-1 chemotherapy postoperatively for a period of 1 year and has been regularly attending our clinic for follow-up examination with CT every 6 months. The patient is still alive without any evidence of local recurrence or metastatic disease 7 years after the initial surgery.

### Discussion

Chemotherapy is a standard treatment for stage IV gastric cancer, but the effect remains unsatisfactory. In the present report, we have experienced a rare case achieved a pCR by chemotherapy with SP. So far, various regimens such as SP, capecitabine + oxaliplatin (XELOX), and capecitabine + cisplatin (XP) + trastuzumab were used for stage IV gastric cancer. Nowadays, several molecular targets have been developed, showing survival benefits to patients with stage IV gastric cancer [14, 15]. However, most of these chemotherapeutic regimens have a low potential for achieving a complete response (Table 1) [16–18]. Regarding to the impact of a pCR on the long-term survival of patients with gastric cancer, Cho et al. demonstrated that the overall survival and recurrence-free survival rates at 3 and 5 years were 96 and 85 and 91 and 75%, respectively [19]. However, this study included patients with various stages of gastric cancer (resectable stage 15/22; unresectable stage 7/22); not all patients were in stage IV. In our case, the patient is still alive with no evidence of recurrence or metastatic disease 7 years after the initial surgery. Therefore, a pCR induced by chemotherapy could be associated with long-term survival.

**Table 1:** Complete response rate in cases after chemotherapy for advanced or metastatic gastric cancer

First author	Year	Clinical trial	Regimen	Enrollment number	CR rate	MST
					(%)	(Month)
Unresectable stage						
A Ohtsu [7]	2003	JCOG 9205	5-FU + CDDP	105	0	7.3
K Yamaguchi [9]	2006	–	S-1 + DTX	46	4.3	14
W Koizumi [4]	2008	SPIRITS trial	S-1 + CDDP	87	1.1	13
Bang Y J [16]	2010	ToGA trial	XP + trastuzumab	294	5.4	13.8
Resectable stage						
T Yoshikawa [17]	2009	JCOG 0001	CPT-11 + CDDP	49	2.0^a^	14.6
Y Iwasaki [18]	2013	JCOG 0210	S-1 + CDDP	47	2.1^a^	17.3

*MST* median survival time, *CDDP* cisplatin, *DTX* docetaxel, *CPT-11* irinotecan

^a^ *pCR* pathological complete response

For the decision regarding treatment strategy, an accurate diagnosis is highly essential. Recently, Yoshida et al. proposed a new classification and therapeutic approaches of stage IV gastric cancer [20]. Actually, it is difficult to diagnose peritoneal dissemination prior to treatment. Reportedly, the sensitivity of CT for peritoneal dissemination of gastric cancer was 43–77%, with a specificity of 82–92% [21–23]. The accuracy of positron emission tomography (PET) for peritoneal dissemination was demonstrated 89%, with a sensitivity and specificity of 35–63 and 89–99%, respectively [21–23]. Kawanaka et al. recently indicated high accuracy for detecting peritoneal dissemination with the combination of CT and PET [24]. Therefore, the combination of CT and PET may be useful for diagnosis of peritoneal dissemination. On the other hand, perioperative staging laparoscopy has often been used to detect occult peritoneal metastases in gastrointestinal cancers [25]. In the present case, we did not perform staging laparoscopy prior to chemotherapy because we detected multiple nodules enhanced inhomogeneously in extra gastric area, which suspected the presence of peritoneal dissemination.

Previous reports have demonstrated that the best timing for the operation after chemotherapy was after 2 or 4–6 cycles [20, 26]. Although it is difficult to decide the best timing, former reports suggested that partial response after chemotherapy may be a good indicator for surgery. In this case, we chose surgical treatment after three courses of chemotherapy because of good response. Surgery after chemotherapy is considered to be complicated because of the presence of bulky tumor or post-chemotherapy compared to surgery without chemotherapy. There might be increased blood loss due to prolonged operation time because of fibrotic changes, adherence to adjacent organs, and a wet operative field which may be caused by chemotherapy. Previous reports have demonstrated that the incidence of complications is not significantly higher than that in patients who underwent conventional radical surgery for gastric cancer [17, 27]. Indeed, in our case, the postoperative course of the patient was uneventful. In the future, the morbidity and mortality in such patients who underwent additional surgery should be evaluated using well-designed clinical trial.

## Conclusions

We encountered a rare case achieved a pCR with advanced gastric cancer treated by standard regimen (SP). It is suggested that a pCR induced by chemotherapy and potentially curative resection after intensive chemotherapy could enhance the probability of longer survival.

## Abbreviations

5-FU: 5-Fluorouracil
CT: Computed tomography
pCR: Pathological complete response
PET: Positron emission tomography
RECIST: Response Evaluation Criteria in Solid Tumors
S-1: Tegafur-gimeracil-oteracil potassium

## References

[1] Nashimoto A, Akazawa K, Isobe Y, Miyashiro I, Katai H, Kodera Y (2013). Gastric cancer treated in 2002 in Japan: 2009 annual report of the JGCA nationwide registry. Gastric Cancer.

[2] Japanese Gastric Cancer Association (2016). Japanese gastric cancer treatment guidelines 2014 (ver. 4). Gastric Cancer.

[3] Ajani JA, Bentrem DJ, Besh S, D’Amico TA, Das P, Denlinger C (2013). Gastric cancer, version 2.2013: featured updates to the NCCN guidelines. J Natl Compr Canc Netw.

[4] Koizumi W, Narahara H, Hara T, Takagane A, Akiya T, Takagi M (2008). S-1 plus cisplatin versus S-1 alone for the first-line treatment for advanced gastric cancer (the SPIRITS trial): a phase III trial. Lancet Oncol.

[5] Cutsem EV, Moiseyenko VM, Tjulandin S, Majlis A, Constenla M, Boni C (2006). Phase III study of docetaxel with cisplatin and 5-fluorouracil as first line therapy for advanced gastric cancer: a report of the V325 study group. J Clin Oncol.

[6] Cunningham D, Starling N, Rao S, Iveson T, Nicolson M, Coxon F (2008). Capecitabine and oxaliplatin for advanced esophagogastric cancer. N Engl J Med.

[7] Ohtsu A, Shimada Y, Shirao K, Boku N, Hyodo I, Saito H (2003). Randomized phase III trial of fluorouracil alone versus fluorouracil plus cisplatin versus uracil and tegafur plus mitomycin in patient with unresectable, advanced gastric cancer: the Japan Clinical Oncology Group Study (JCOG9205). J Clin Oncol.

[8] Boku N, Yamamoto S, Fukuda H, Shirao K, Doi T, Sawaki A (2009). Fluorouracil versus combination of irrinotecan plus cisplatin versus S-1 in metastatic gastric cancer: a randomized phase 3 study. Lancet Oncol.

[9] Yamaguchi K, Shimamura T, Hyodo I, Koizumi W, Doi T, Narahara H (2006). Phase I/II study of docetaxel and S-1 in patients with advanced gastric cancer. Br J Cancer.

[10] Wang Y, Yu YY, Li W, Feng Y, Hou J, Ji Y (2014). A phase II trial of Xeloda and oxaliplatin (XELOX) neo-adjuvant chemotherapy followed by surgery for advanced gastric cancer patients with para-aortic lymph node metastasis. Cancer Chemother Pharmacol.

[11] Okabe H, Ueda S, Obama K, Hosogi H, Sakai Y (2009). Induction chemotherapy with S-1 plus cisplatin followed by surgery for treatment of gastric cancer with peritoneal dissemination. Ann Surg Oncol.

[12] Suzuki T, Tanabe K, Taomoto J, Yamamoto H, Tokumoto N, Yoshida K (2010). Preliminary trial of adjuvant surgery for advanced gastric cancer. Oncol Lett.

[13] Eisenhauer EA, Therasse P, Bogaerts J, Schwartz LH, Sargent D, Ford R (2009). New response evaluation criteria in solid tumours: revised RECIST guideline (version 1.1). Eur J Cancer.

[14] Fuchs CS, Tomasek J, Yong CJ, Dumitru F, Passalacqua R, Goswami C (2014). Ramucirumab monotherapy for previously treated advanced gastric or gastro-oesophageal junction adenocarcinoma (REGARD): an international, randomised, multicenter, placebo-controlled, phase 3 trial. Lancet.

[15] Wilke H, Muro K, Van Cutsem EV, Oh SC, Bodoky G, Shimada Y (2014). Ramucirumab plus paclitaxel versus placebo plus paclitaxel in patients with previously treated advanced gastric or gastro-oesophageal junction adenocarcinoma (RAINBOW): a double-blind, randomised phase 3 trial. Lancet Oncol.

[16] Bang YJ, Cutsem EV, Feyereislova A, Chung HC, Shen L, Sawaki A (2010). Trastuzumab in combination with chemotherapy versus chemotherapy alone for treatment of HER2-positive advanced gastric or gastro-oesophageal junction cancer (ToGA): a phase 3, open-label, randomised controlled trial. Lancet.

[17] Yoshikawa T, Sasako M, Yamamoto S, Sano T, Imamura H, Fujitani K (2009). Phase II study of neoadjuvant chemotherapy and extended surgery for locally advanced gastric cancer. Br J Surg.

[18] Iwasaki Y, Sasako M, Yamamoto S, Nakamura K, Sano T, Katai H (2013). Phase II study of preoperative chemotherapy with S-1 and cisplatin followed by gastrectomy for clinically resectable type 4 and large type 3 gastric cancers (JCOG0210). J Surg Oncol.

[19] Cho H, Nakamura J, Asaumi Y, Yabusaki H, Sakon M, Takasu N (2015). Long-term survival outcomes of advanced gastric cancer patients who achieved a pathological complete response with neoadjuvant chemotherapy: a systematic review of the literature. Ann Surg Oncol.

[20] Yoshida K, Yamaguchi K, Okumura N, Tanahashi T, Kodera Y (2016). Is conversion therapy possible in stage IV gastric cancer: the proposal of new biological categories of classification. Gastric Cancer.

[21] Turlakow A, Yeung HW, Salmon AS, Macapinlac HA, Larson SM (2003). Peritoneal carcinomatosis: role of ^18^F-FDG PET. J Nucl Med.

[22] Lim JS, Kim MJ, Yun MJ, Oh YT, Kim JH, Hwang HS (2006). Comparison of CT and ^18^F-FDG PET for detecting peritoneal metastasis on the preoperative evaluation for gastric carcinoma. Korean J Radiol.

[23] Pfannenberg C, Konigstrainer I, Aschoff P, Oksuz MO, Zieker D, Beckert S (2009). ^18^F-FDG-PET/CT to select patients with peritoneal carcinomatosis for cytoreductive surgery and hyperthermic intraperitoneal chemotherapy. Ann Surg Oncol.

[24] Kawanaka Y, Kitajima K, Fukushima K, Mouri M, Doi H, Oshima T (2016). Added value of pretreatment ^18^F-FDG PET/CT for staging of advanced gastric cancer: comparison with contrast-enhanced MDCT. Eur J Radiol.

[25] Ishigami S, Uenosono Y, Arigami T, Yanagita S, Okumura H, Uchikado Y (2014). Clinical utility of perioperative staging laparoscopy for advanced gastric cancer. World J Surg Oncol.

[26] Yoshikawa T, Tanabe K, Nishikawa K, Ito Y, Matsui T, Kimura Y (2014). Induction of a pathological complete response by four courses of neoadjuvant chemotherapy for gastric cancer: early results of the randomized phase II COMPASS trial. Ann Surg Oncol.

[27] Fujitani K, Ajani JA, Crane CH, Feig BW, Pisters PW, Janjan N (2007). Impact of induction chemotherapy and preoperative chemoradiotherapy on operative morbidity and mortality in patients with locoregional adenocarcinoma of the stomach or gastroesophageal junction. Ann Surg Oncol.

